# P074: Incidence of carbapenemase-producing Klebsialla pneumoniae at the University Hospital of Salloul (Sousse-Tunisia)

**DOI:** 10.1186/2047-2994-2-S1-P74

**Published:** 2013-06-20

**Authors:** O Bouallègue, N Jaidane, L Dhidah, Aziza Masoudi, Nourredine Boujaafar

**Affiliations:** 1 Microbiology Laboratory, Hospital of Sahloul, Sousse, Tunisia; 2 Surgical ICU, Sousse, Tunisia; 3 Hospital Hygiene Department, Hospital of Sahloul, Sousse, Tunisia

## Introduction

Recently, multidrug resistant *Klebsiella pneumoniae* especially Carbapenemase-producing has been identified in Tunisia and becoming an epidemic emergent widely spread phenomenon.

## Objectives

To describe the epidemiologic profile of nosocomial infections caused by K. pneumoniae.

## Methods

A prospective surveillance study was performaed at a university hospital of Sahloul (Sousse-Tunisia) from july 2011 to march 2012. K. pneumoniae isolates were identified in the clinical laboratory by biochemical tests and the Analytical Profile index procedure (API 20-NE -Biomérieux, France). Antimicrobial susceptibility testing was performed by standardized methods recommended by the National Committee of Clinical Laboratory Standards. Occurrence of beta-lactamases was detected by PCR amplification and sequencing of ESBL genes (*bla*TEM, *bla*SHV, *bla*CTX-M) and carbapenemase genes (*bla*OXA-48).*ERIC*-*PCR*genotyping were used to assess genetic heterogeneity between the isolates. MIC determinations for carbapenems were performed by Etest (bioMérieux).

## Results

Forty three strains were collected from 43 patients admitted in the ICU and the urology service. The repeat isolates were excluded from the study. All the patients in our study have had indwelling intravascular devices or were exposed to invasive procedures. During the study period two epidemic periods was declared: the first one occurred between august and September 2011 and the second between December and February 2012. Antimicrobial susceptibility pattern of all clinical isolates revealed four different profiles based on sensivity patterns against fosfomycin, colistin, co-trimxazole, tygecyclin and aminosides. PCR and sequencing analysis revealed that the isolates harbored the *bla*CTX gene, the *bla*SHV and the *bla*OXA-48 gene.

## Conclusion

There is a serious need to accentuate on the rational use of antimicrobials and strictly adhere to the concept of the “reserve drug” to minimize the misuse of available antimicrobials. In addition regular antimicrobial susceptibility surveillance, knowledge and its application is essential to reduced current drug resistance rate in hospital as well as in community, in addition to the implementation of basic hygiene precautions.

## Disclosure of interest

None declared

